# Defect-Repairable Latent Feature Extraction of Driving Behavior via a Deep Sparse Autoencoder

**DOI:** 10.3390/s18020608

**Published:** 2018-02-16

**Authors:** Hailong Liu, Tadahiro Taniguchi, Kazuhito Takenaka, Takashi Bando

**Affiliations:** 1 The Graduate School of Information Science and Engineering, Ritsumeikan University, Kusatsu, Shiga 525-8577, Japan; 2 Research Fellow (DC) with the Japan Society for the Promotion of Science, Tokyo 102-0083, Japan; 3 The College of Information Science and Engineering, Ritsumeikan University, Kusatsu, Shiga 525-8577, Japan; taniguchi@em.ci.ritsumei.ac.jp; 4 The Corporate R&D Div.1, Sensing System R&D Dept., DENSO CORPORATION, Aichi 448-8661, Japan; kazuhito_takenaka@denso.co.jp; 5 The DENSO International America, Inc., San Jose, CA 95110, USA; takashi_bando@denso-diam.com

**Keywords:** feature extraction, defects repairing, deep learning, driving behavior analysis

## Abstract

Data representing driving behavior, as measured by various sensors installed in a vehicle, are collected as multi-dimensional sensor time-series data. These data often include redundant information, e.g., both the speed of wheels and the engine speed represent the velocity of the vehicle. Redundant information can be expected to complicate the data analysis, e.g., more factors need to be analyzed; even varying the levels of redundancy can influence the results of the analysis. We assume that the measured multi-dimensional sensor time-series data of driving behavior are generated from low-dimensional data shared by the many types of one-dimensional data of which multi-dimensional time-series data are composed. Meanwhile, sensor time-series data may be defective because of sensor failure. Therefore, another important function is to reduce the negative effect of defective data when extracting low-dimensional time-series data. This study proposes a defect-repairable feature extraction method based on a deep sparse autoencoder (DSAE) to extract low-dimensional time-series data. In the experiments, we show that DSAE provides high-performance latent feature extraction for driving behavior, even for defective sensor time-series data. In addition, we show that the negative effect of defects on the driving behavior segmentation task could be reduced using the latent features extracted by DSAE.

## 1. Introduction

Driving behavior can be measured as multi-dimensional time-series data, i.e., the driving behavior data generated by a variety of sensors in a vehicle, e.g., the speed of the wheels, steering angle, and accelerator opening rate. However, these multi-dimensional time-series data often include redundant information, i.e., a variety of sensor time-series data could present the same feature of driving behavior. A simple example is that the sensor time-series data of the speed of the wheels and meter readings of the velocity, as well as the engine speed can all present the velocity of the vehicle, even though these data have been obtained from different sensors or different sensing process involving different degrees of noise, artifacts, and influences from other variables, e.g., a gear ratio. Meanwhile, time-series data measured by a sensor can also be generated by fusing the original one-dimensional time-series data. For example, the sensor time-series data of the yaw rate can be regarded as data generated by fusing latent time-series data related to the velocity and the change in the driving direction (steering). For that reason, we can assume that some sensor time-series data are generated by fusing a variety of the latent low-dimensional time-series data. Redundant information can be expected to complicate the data analysis, e.g., more factors need to be analyzed, and more calculations are needed; even varying levels of redundancy can influence the results of the analysis. Therefore, reducing the redundant information besides retaining the latent low-dimensional time-series data is a main task for this study. If the low-dimensional time-series data can be extracted from the driving behavior data, then we can use them in a variety of driving behavior analyses, e.g., recognition, prediction, and visualization.

A sensor, however, introduces noise, outliers, and defects into the time-series data by interacting with the environment and other sensors when a failure of the sensor occurs. Such defects in the time-series may negatively affect the extracted low-dimensional time-series data, because incorrect values are often measured in a period of frames. In addition, those defects also destroy the relevancy between data before and after them, e.g., the context of driving behaviors. For example, if we were to use a Hidden Markov model (HMM) for driving behavior analysis, defective data would result in the rupture of the latent Markov chain and produce unsatisfactory results. Thus, an important and challenging problem emerges from the above discussion: how to extract the low-dimensional time-series data of driving behavior. To address this problem, we need a method that is able to automatically extract low-dimensional time-series data from the measured multi-dimensional driving behavior data even when the data include redundant dimensions. Moreover, this method should also reduce the negative effect of defects in sensor time-series data on the extracted low-dimensional time-series data.

In this study, we start from the premise that defects can be detected because there are many existing methods that can detect outliers and defects [1,2,3]. We focus on and propose a method to extract low-dimensional time-series data of driving behavior with defects along with repairing the defective data. The proposed method is a deep learning method using a deep sparse autoencoder (DSAE), which can also repair defects introduced in measured driving behavior data. The DSAE is piled up by a number of autoencoder that reduces dimensionality by finding redundancy and detecting repetitive structures of data automatically [4]. The low-dimensional time-series data can be extracted as time-series of the latent features of a neural network, i.e., the activation patterns on hidden layers in the DSAE. The extracted time-series of latent features can represent driving behavior data. The advantage of this method is that DSAE only needs to be trained once and it can accomplish two tasks: extracting time-series of latent features and repairing defects in sensor time-series data. We reported brief descriptions and preliminary results of this study in [5,6]. In this paper, we present a complete description and experimental evaluation of our method and demonstrate the validity of our proposed method based on several experiments. We also propose using DSAE with a back propagation method (DSAE-BP) to repair the defects in sensor time-series data. The main contributions of this paper are as follows.

- We show that DSAE can extract highly correlated low-dimensional time-series of latent features by reducing various degrees of redundancy in different multi-dimensional time-series data of driving behavior.
- We verify that DSAE can reduce the negative effect of defects on the extracted time-series of latent features by repairing the defective sensor time-series data using a BP method.
- We find that the time-series of latent features extracted from the repaired time-series sensor data by DSAE have segmentation results similar to those of non-defective sensor time-series data.

As a result, we show that DSAE can be used as a highly useful and defect-repairable feature extraction method for driving behavior.

The remainder of this paper is organized as follows. Section 2 presents background material related to this research. Section 3 describes the proposed method including the feature extraction process and defect repair process. Section 4 and Section 5 verify the effectiveness of the proposed method in terms of feature extraction and defect repair. Section 6 provides an application of the proposed method to driving behavior segmentation. Section 7 discusses the advantages and limitations of proposed method. Section 8 concludes this paper.

## 2. Background

This section presents background material on three aspects of the research: feature extraction methods for driving behavior analysis, a feature extraction method by deep learning for intelligent vehicles, and defect repair for driving behavior analysis.

### 2.1. Feature Extraction for Driving Behavior Analysis

In most studies about driving behavior analysis, the feature vectors that are regarded as input data of the analysis method were selected manually from the measured sensor data [7,8,9] and designed manually [10]. For example, Taniguchi et al. selected the velocity, steering angle, brake pressure, accelerator position, and designed the temporal difference between the velocity and steering angle as input data of the HMM for driving behavior prediction [8]. Li et al. designed a feature for drunk driving detection by using the respective slopes of the lateral position of the vehicle, steering angle, and time interval [10]. However, we consider the feature design and selection to rely on expert experience, and finding an appropriate way of feature representations for each dataset is often difficult, especially for driving behaviors that are obtained from a driver–vehicle system including a large number of variables, e.g., the impact of the external environment on the driver, the driver’s driving intention, and the vehicle dynamics. Compared with manual selection and manual feature design, we can apply an automatic feature extraction method for obtaining low-dimensional time-series data that can represent multi-dimensional data from many sensors. Therefore, we selected an unsupervised feature extraction method that can extract low-dimensional time-series of latent features from multi-dimensional sensor time-series data automatically.

Principal component analysis (PCA) is extensively used for unsupervised feature extraction [11]. PCA can project data into a low-dimensional feature space and find the principal components by maximizing the variance of the data in the projected feature space. Chatzigiannakis et al. proposed using PCA to detect traffic incidents, e.g., traffic accidents and low-speed vehicle flow [12]. Meanwhile, Calderó-Bardají et al. also used PCA to extract the latent features of electroencephalography and used a support vector machine to detect the steering direction prior [13]. In addition, Lin et al. proposed using an independent component analysis (ICA) method for predicting the driver’s state of drowsiness [14]. The ICA can extract independent latent features from multivariate signals [15]. This method assumes that the measured signals are generated via linear transformations from source signals. However, ICA is very sensitive to outliers when it uses the kurtosis measure as an optimization criterion [16]. Therefore, ICA is not robust for extracting latent features of driving behavior when the defects were detected.

PCA and ICA cannot use a nonlinear transformation to extract time-series of latent features from driving behavior data because vehicle dynamics and human driving behavior have nonlinear properties. This problem could be solved by using kernel principal components analysis (KPCA) [17]. For example, Zhao et al. used KPCA to extract latent features for identifying a driver’s mental fatigue. This method had a higher accuracy rate than PCA [18]. KPCA uses a nonlinear kernel function that involves a nonlinear transformation to map the data onto a high-dimensional space. However, when there is a large volume of driving behavior data, the computational cost of the KPCA method is high, because the kernel method must compute a gram matrix in $R^{N\times N}$, where *N* is the total number of frames of data. In this paper, we propose using DSAE for extracting the time-series of latent features for driving behavior. DSAE has the advantages of being able to process both nonlinear data and large datasets.

### 2.2. Feature Extraction by Deep Learning for Intelligent Vehicles

In recent years, feature extraction methods employing deep learning approaches have attracted considerable attention. Deep learning methods use neural networks with a deep structure, i.e., they have three or more layers. Deep learning has previously been used in the field of intelligent vehicles for a variety of tasks. For example, Dong et al. proposed a regularized network that includes a stacked recurrent numerous network (RNN) and an autoencoder to use the GPS signal to analyze driving styles [19]. Hori et al. used a long short-term memory RNN to detect the confusion state of a driver [20]. Although the above two studies included feature extraction for time-series analysis, those features were extracted by supervised learning models for respective tasks. We used an unsupervised learning method for feature extraction in this study, because, if the time-series of latent features extracted via an unsupervised learning method are able to represent driving behavior, they can support varied tasks including tasks based on the unsupervised method, e.g., time-series segmentation [21] and data visualization [22].

Meanwhile, we consider that the feature extraction for driving behaviors in the lower layer of the deep learning model can be used to access the latent features of relatively simple driving behaviors, e.g., turning left and accelerating forward. As the number of layers increases, the latent features in the lower layer will be fused to form latent features that can represent more complex driving behaviors, e.g., turning left while accelerating. For example, [22] showed that a visualization result based on time-series of latent features extracted by using a deep learning method, i.e., DSAE, could determine complex driving behaviors more effectively than by using a feature extraction methods with a shallow structure, e.g., a sparse autoencoder (SAE), PCA, ICA, and KPCA. However, [22] evaluated the performance of DSAE in a task of driving behavior visualization. In this paper, we rigorously verified the capability of DSAE to extract the time-series of latent features of driving behavior.

### 2.3. Defect Repair for Driving Behavior Analysis

The presence of noise, outliers, and defective input data has a negative effect on many kinds of analyses. In the field of intelligent vehicles, many studies have proposed detection methods for outliers and defects. For example, Tagawa et al. proposed defect detection by using a structured denoising autoencoder, which they effectively applied to real driving data [1]. Krishnaswami et al. used a parity equation residual generation method to detect defective sensor information for diagnosing engine faults [2]. Malhotra et al. used long short-term memory networks to detect an anomaly in time-series, and they reported that this method had a promising result on multi-sensor engine data [3]. Therefore, we started with the premise that defects and outliers exist in driving behavior data.

To repair those outliers and defects, Liu et al. used a windowing process as the smoothing process to smooth noise in a pre-processing step of driving behavior visualization [22]. However, a windowing process cannot easily solve the problems caused by outliers and defects. Instead, a windowing process allows outliers and defects to have a negative effect on the time axis. Kaneda et al. used a robust Kalman filter to remove the outliers of vehicle control [23]. Jang alleviated traffic congestion by using a modified median filter to remove outliers of dedicated short-range communications probe data in an advanced traveler information system [24]. Usually, measurements do not only frequently contain outliers, but the defects in sensor time-series data also include incorrect measurements over a period of frames. The above two methods are unable to repair the data for a period in which many defects are found. Therefore, in this study, our approach to repairing a period of defects is to use prediction by a trained DSAE. The advantage of our method is that the DSAE can extract time-series of latent features and repair defects in sensor time-series data by training the DSAE model only one time.

Two previous studies used a deep learning method to repair defects in the field of intelligent transportation systems. Duan et al. used stacked denoising autoencoders for the imputation of defective traffic data [25]. Ku et al. also used stacked denoising autoencoders that were constructed for road segments to extract their spatial-temporal relationships to use them for imputing defects [26]. The above two studies both used a deep learning method based on a stacked denoising autoencoder. Because the denoising autoencoder added some noise and defects to the training data when the model was being trained, both of the above studies led to the development of a model with a generalization of noise and defects. In addition, it should be noted that these models [25,26] focused on simply repairing defects in the input data. In contrast, the purpose of this study is to extract time-series of latent features that can represent driving behaviors, even those containing defects, in the measured sensor time-series data.

## 3. Proposed Method

**Figure 1:**
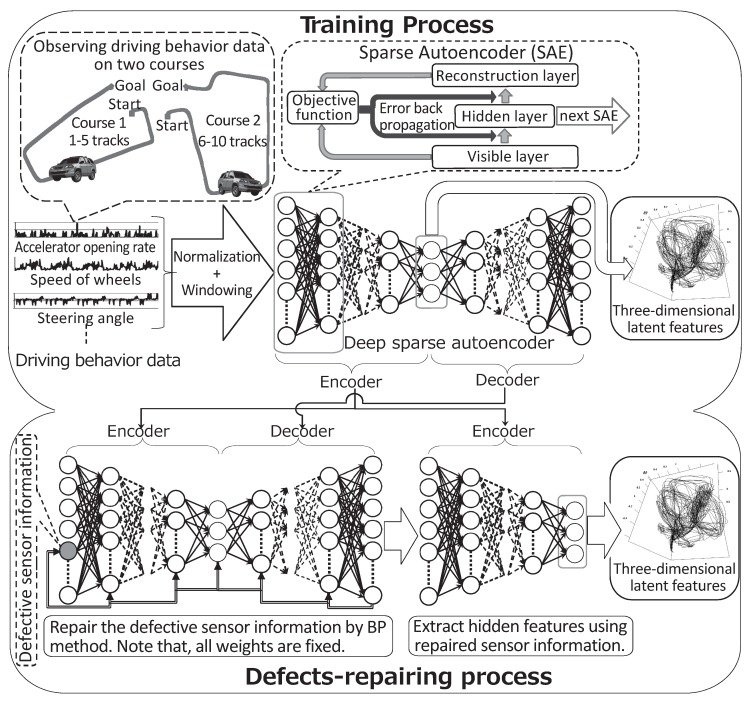
Feature extraction and defect-repairing processes of the deep sparse autoencoder (DSAE), which repairs defects in sensor time-series data by using a back propagation (BP) method.

We propose the use of the deep learning method DSAE to extract the time-series of latent features for driving behavior. DSAE can also reduce the negative effects on feature extraction when the sensor time-series data contain defects, which starts from the premise that defects can be detected. Our proposed method is illustrated in Figure 1, which shows two steps: the training process and defect-repairing process. The training process trains the DSAE with non-defective sensor time-series data of driving behavior. The defect-repairing process repairs defects in the sensor time-series data and extracts the time-series of latent features by using the trained DSAE.

### 3.1. Training Process

In the training process, the DSAE is trained to extract a low-dimensional time-series latent feature from driving behavior data. Before data are input into the DSAE, we use two pre-processing steps: normalization and windowing processing. Normalization is used to deal with dimensionless quantity because the unit of each kind of sensor time-series data of driving behavior is different. We define the measured driving behavior data as $Y\in R^{D_{Y}\times N_{Y}}$, where each dimension represents one kind of sensor time-series data and where $D_{Y}$ is the dimensionality and $N_{Y}$ is the quantity of data (frames) in Y. The *t*-th datum is defined as $y_{t}={\left(y_{t,1},y_{t,2},\ldots ,y_{t,D_{Y}}\right)}^{T}\in R^{D_{Y}}.$ Note that, the activation function we employ in the DSAE uses a hyperbolic tangent function of which the output range is (−1,1). However, the range of the measured driving behavior data in the dataset Y is (−∞,∞). To reconstruct the input data with tanh, we independently normalize each dimension of the input data into (−1,1) by using the maximum and minimum values. Therefore, the *t*-th normalized datum is expressed as $x_{t}={\left(x_{t,1},x_{t,2},\ldots ,x_{t,D_{Y}}\right)}^{T}\in R^{D_{Y}},$ where

(1) $$\begin{array}{c} x_{t,d}=2\left(\frac{y_{t,d}-\min\left(y_{1:N_{y},d}\right)}{\max\left(y_{1:N_{y},d}\right)-\min\left(y_{1:N_{y},d}\right)}\right)-1 \end{array}$$

In addition, to extract the latent features with a property of time-series, a windowing process enables the DSAE to fully connect a period of time steps of driving behavior data in the input layer.

In the windowing process, we aggregate the normalized data with a slide window to convert the data of *w* frames into a vector. Therefore, the *t*-th frame of the windowing time-series data $h_{t}^{\left(1\right)}$ is expressed as $h_{t}^{\left(1\right)}={\left(x_{t-w+1}^{T},x_{t-w+2}^{T},\ldots ,x_{D_{V}}^{T}\right)}^{T}\in R^{D_{H}^{\left(1\right)}},$ where $D_{H}^{\left(1\right)}=w\times D_{Y},t\geq w$. We notate these time-series data as $h_{t}^{\left(1\right)}$ with superscript (1) because the time-series data is used as an input for the first layer in the network of the DSAE. Finally, we obtain the windowing time-series data, which are $H^{\left(1\right)}=\left\{h_{1}^{\left(1\right)},h_{2}^{\left(1\right)},\ldots ,h_{N_{H^{\left(1\right)}}}^{\left(1\right)}\right\}\in R^{D_{H^{\left(1\right)}}\times N_{H^{\left(1\right)}}}$, when the slide window moves along the time axis in a step-by-step manner. The frames in $H^{\left(1\right)}$ are $N_{H^{\left(1\right)}}=N_{Y}-w+1$.

The DSAE is a deep neural network with a symmetrical structure and an odd number of layers. The DSAE extracts time-series of latent features by minimizing the error between the input time-series data and decoded time-series data through an encoding–decoding process. The DSAE is presented diagrammatically in the upper part of Figure 1. If DSAE has *L* layers ($L\in \{2n+1|n\in Z^{+}\}$), then the layers from the first layer to the middle layer, notated as the m-th layer, where m=(L+1)/2, are the encoding layers. Meanwhile, the decoding layers are those from the *m*-th layer to the *L*-th layer. The time-series data of the middle layer (the *m*-th layer) of the DSAE are the time-series of the latent features of the input data.

The mappings between all pairs of adjacent layers can be expressed by the same equation. For example, the *t*-frame data $h_{t}^{\left(l\right)}$ of the *l*-th layer are mapped to the l+1-th layer via

(2) $$\begin{array}{ccc} h_{t}^{\left(l+1\right)} & = & f\left(W^{\left(l\right)}h_{t}^{\left(l\right)}+b^{\left(l\right)}\right)\in R^{D_{H}^{\left(l\right)}}, \end{array}$$

in which the function *f* is an activation function of DSAE and $l\in Z^{+}$, l<L. In this study, we selected a hyperbolic tangent function f(·)=tanh(·) as the activation function because the hyperbolic tangent function outperforms the sigmoid function and radial bases function [27]. Meanwhile, $W^{\left(l\right)}\in R^{D_{H}^{\left(l\right)}\times D_{H}^{\left(l+1\right)}}$ and $b^{\left(l\right)}\in R^{D_{H}^{\left(l\right)}}$ are the weight matrix and bias vector. $D_{H}^{\left(l\right)}$ is the dimensionality of the vector of the *l*-th layer. The objective function of a DSAE with *L* layers is

(3) $$\begin{array}{ccc} J(\Phi ) & = & \frac{1}{2N_{V}}\sum_{t=1}^{N_{V}}\left|\right|h_{t}^{\left(L\right)}-h_{t}^{\left(1\right)}{\left|\right|}_{2}^{2}+\frac{\alpha }{2}\sum_{l=1}^{L-1}\left|\right|W^{\left(l\right)}{\left|\right|}_{2}^{2}+\beta \sum_{i=1}^{D_{H}^{\left(l\right)}}\mathrm{KL}(\omega ||{\bar{h}}_{i}^{\left(m\right)}), \end{array}$$

where $\Phi =\{W^{\left(1\right)},\ldots ,W^{\left(L-1\right)},b^{\left(1\right)},\ldots ,b^{\left(L-1\right)}\}$. The first term is an error term that shows the square error between the input data and decoded data. The second term is a penalty term that limits the elements of all the weights $W^{\left(l\right)}$ with the L2 norm to prevent over-fitting. The parameter α can control the strength of the penalty term. The third term is a sparse term to ensure data sparsity in the m-th layer. It is because this term would enable more obvious features to be obtained. The sparse term is a Kullback–Leibler divergence between two Bernoulli random variables with ω and ${\bar{h}}_{i}^{\left(m\right)}$ [28].

(4) $$\begin{array}{ccc} \mathrm{KL}(\omega ||{\bar{h}}_{i}^{\left(m\right)}) & = & \omega \log\frac{\omega }{{\bar{h}}_{i}^{\left(m\right)}}+\left(1-\omega \right)\log\frac{1-\omega }{1-{\bar{h}}_{i}^{\left(m\right)}}, \end{array}$$

(5) $$\begin{array}{ccc} {\bar{h}}_{i}^{\left(m\right)} & = & \frac{1}{2}\left(1+\frac{1}{N_{V}}\sum_{t=1}^{N_{V}}h_{t,i}^{\left(m\right)}\right), \end{array}$$

where ω is the sparsity target of the hidden layer and $h_{t,i}^{\left(m\right)}$ is the *i*-th element of $h_{t}^{\left(m\right)}$. The value of β can be used to control the strength of the sparse term. If the sparse term was minimized, then ${\bar{h}}_{i}^{\left(m\right)}$ will be close to ω.

Finally, we use a back propagation (BP) method [29] to minimize the objective function for training the DSAE. The BP method performs partial differentiations of the weight matrices and biases for the objective function Equation (3) via a chain rule. Therefore, the equations to update the weight matrix $W^{\left(l\right)}$ and the bias vector $b^{\left(l\right)}$ between the *l*-th and (l+1)-th layers are:

(6) $$\begin{array}{ccc} {W^{+}}^{\left(l\right)} & \leftarrow & W^{\left(l\right)}-\lambda^{\left(l\right)}\frac{\partial J(\Phi )}{\partial W^{\left(l\right)}},\\ & = & W^{\left(l\right)}-\lambda^{\left(l\right)}\left(\frac{1}{N_{v}}\sum_{t=1}^{N_{v}}h_{t}^{\left(l\right)}\gamma_{t}^{\left(l\right)}+\alpha W^{\left(l\right)}\right), \end{array}$$

(7) $$\begin{array}{ccc} {b^{+}}^{\left(l\right)} & \leftarrow & b^{\left(l\right)}-\lambda^{\left(l\right)}\frac{\partial J(\Phi )}{\partial b^{\left(l\right)}},\\ & = & b^{\left(l\right)}-\lambda^{\left(l\right)}\left(\frac{1}{N_{v}}\sum_{t=1}^{N_{v}}\gamma_{t}^{\left(l\right)}\right), \end{array}$$

where ${\left(\cdot \right)}^{+}$ signifies the value that has been updated. The vector $\gamma_{t}^{\left(l\right)}\in R^{D_{H}^{\left(l\right)}}$ is calculated via

(8) $$\begin{array}{c} \gamma_{t}^{\left(l\right)}=\left\{\begin{array}{cc} -D^{\left(l+1\right)}\left(h_{t}^{\left(1\right)}-h_{t}^{\left(L\right)}\right) & \left(l=L-1\right)\\ D^{\left(l+1\right)}\left(W^{\left(l+1\right)}\gamma_{t}^{\left(l+1\right)}\right)+\beta \epsilon & \left(l=m-1\right)\\ D^{\left(l+1\right)}\left(W^{\left(l+1\right)}\gamma_{t}^{\left(l+1\right)}\right) & (\mathrm{others}), \end{array}\right. \end{array}$$

where $D^{\left(l\right)}$ is a diagonal matrix shown by

(9) $$\begin{array}{c} D^{\left(l\right)}=\mathrm{diag}\left(1-{\left(h_{t,1}^{\left(l\right)}\right)}^{2},1-{\left(h_{t,2}^{\left(l\right)}\right)}^{2},\ldots ,1-{\left(h_{t,D_{H}^{\left(l\right)}}^{\left(l\right)}\right)}^{2}\right); \end{array}$$

and the *i*-th element of the vector $\epsilon \in R^{D_{H}^{\left(l\right)}}$ is

$$\begin{array}{c} \epsilon_{i}=\frac{1-\omega }{1-{\bar{h}}_{i}^{\left(m\right)}}-\frac{\omega }{{\bar{h}}_{i}^{\left(m\right)}}. \end{array}$$

In the above updating equations, $\lambda^{\left(l\right)}$ is the dynamic learning rate of $W^{\left(l\right)}$ and $b^{\left(l\right)}$. The dynamic learning rate is changed in every iterative cycle via a line search, for which we define the searching distance θ. The dynamic learning rate $\lambda^{\left(l\right)}$ and searching distance θ are initialized by small positive numbers. Therefore, θ is updated by:

(10) $$\begin{array}{c} \theta^{+}=\left\{\begin{array}{cc} -0.5\theta & \left(J^{+}\left(\Phi \right)>J\left(\Phi \right)\right)\\ \theta & \left(J^{+}\left(\Phi \right)<J\left(\Phi \right)\right). \end{array}\right. \end{array}$$

Then, the appropriated dynamic learning rate is ${\lambda^{\left(l\right)}}^{+}=\lambda^{\left(l\right)}+\theta$. The line search is set to terminate when the value of $|J^{+}\left(\Phi \right)-J\left(\Phi \right)|$ becomes smaller than a certain small threshold.

To prevent the weight and bias from converging to inaccurate local optima, we use a procedure known as greedy layer-wise training. We divide the DSAE into a number of SAEs that include three layers. Each SAE is responsible for optimizing a set of weights and bias between every two layers of DSAE. Meanwhile, the SAE also outputs the data in its *m*-th layer as the input data of the next SAE. After using SAE to train all the weights and bias layer by layer, we use those trained weights and bias as initialization for the DSAE, and re-train them through the multi-layer neural network of DSAE.

### 3.2. Defect-Repairing Process

We consider a trained DSAE capable of repairing the defects in sensor time-series data via the encoding-decoding process because it is trained by minimizing the squared error between the input data and reconstructed data, which is the decoded data. In the work presented in this paper, we assume that the defective sensor is given. In [6], we used a DSAE with a fixed-point iterative method (DSAE-FP) to reduce the negative effect of defects on the driving behavior in a segmentation task. In addition, we propose the DSAE with back propagation method (DSAE-BP) in this section.

#### 3.2.1. DSAE-FP

**Figure 2:**
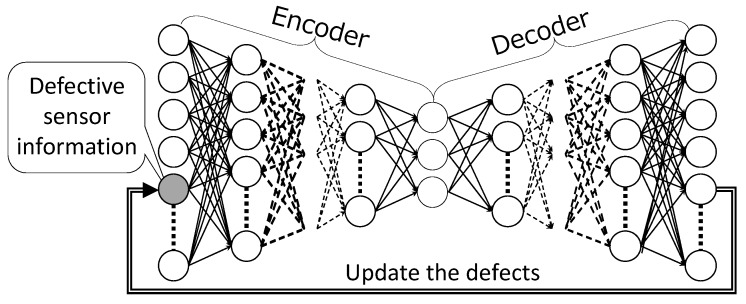
DSAE-FP: Repair the defects in sensor time-series data by updating to the reconstructed time-series data.

The DSAE-FP, which inputs sensor time-series data with defects into the trained DSAE, and outputs the reconstructed time-series data, is illustrated in Figure 2. Then, the defects are updated by the corresponding elements of the reconstructed time-series data. Several iterations of the above steps are necessary for convergence to a fixed point. However, DSAE-FP only uses the potential of data reconstruction to repair the defects in sensor time-series data. Therefore, DSAE-FP would easily cause the defects to converge to different fixed points if the initial defect values were different.

#### 3.2.2. DSAE-BP

**Figure 3:**
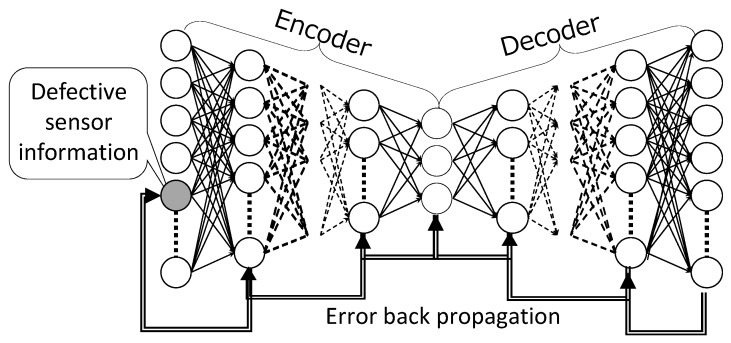
DSAE-BP: Repair the defects in sensor time-series by a BP method. Weights and biases of DSAE are not changed.

An overview of DSAE-BP is shown in Figure 3. The advantage of using the BP method is that it guarantees that the defects are updated in the direction in which the reconstruction error is minimized using the BP method. The reconstruction error of *t*-th time step is

(11) $$\begin{array}{ccc} E{\left(\Phi \right)}_{t} & = & \frac{1}{2}\left|\right|h_{t}^{\left(L\right)}-h_{t}^{\left(1\right)}{\left|\right|}_{2}^{2}. \end{array}$$

We update the defective element $h_{t^{\left(1\right)},d}$ in the sensor time-series data via

(12) $$\begin{array}{c} {\left(h_{t,d}^{\left(1\right)}\right)}^{+}=h_{t,d}^{\left(1\right)}-\psi {\left(\frac{\partial E{\left(\Phi \right)}_{t}}{\partial h_{t}^{\left(1\right)}}\right)}_{d}, \end{array}$$

where ψ is the learning rate and ${\left(\frac{\partial E{\left(\Phi \right)}_{t}}{\partial h_{t}^{\left(1\right)}}\right)}_{d}$ is the *d*-th element of the vector $\frac{\partial E{\left(\Phi \right)}_{t}}{\partial h_{t}^{\left(1\right)}}$. This partial differential is expanded as,

(13) $$\begin{array}{ccc} \frac{\partial E{\left(\Phi \right)}_{t}}{\partial h_{t}^{\left(1\right)}} & = & \frac{1}{2}\frac{\partial E{\left(\Phi \right)}_{t}}{\partial (h_{t}^{\left(1\right)}-h_{t}^{\left(L\right)})}\frac{\partial (h_{t}^{\left(1\right)}-h_{t}^{\left(L\right)})}{\partial h_{t}^{\left(1\right)}}\\ & = & \left(h_{t}^{\left(1\right)}-h_{t}^{\left(L\right)}\right)\frac{\partial (h_{t}^{\left(1\right)}-h_{t}^{\left(L\right)})}{\partial h_{t}^{\left(1\right)}}\\ & = & \left(h_{t}^{\left(1\right)}-h_{t}^{\left(L\right)}\right)-\left(h_{t}^{\left(1\right)}-h_{t}^{\left(L\right)}\right)\frac{\partial h_{t}^{\left(L\right)}}{\partial h_{t}^{\left(1\right)}},\\ \frac{\partial h_{t}^{\left(L\right)}}{\partial h_{t}^{\left(1\right)}} & = & \frac{\partial h_{t}^{\left(L\right)}}{\partial h_{t}^{\left(L-1\right)}}\frac{\partial h_{t}^{\left(L-1\right)}}{\partial h_{t}^{\left(L-2\right)}}\times \ldots \times \frac{\partial h_{t}^{\left(2\right)}}{\partial h_{t}^{\left(1\right)}}, \end{array}$$

through the chain rule. The mapping between each two layers of the DSAE is the same as in Equation (2); therefore, the partial derivative of the *l*-th layer with respect to the (l−1)-th layer can be expressed by

(14) $$\begin{array}{c} \frac{\partial h_{t}^{\left(l\right)}}{\partial h_{t}^{\left(l-1\right)}}=D^{\left(l\right)}W^{\left(l-1\right)}. \end{array}$$

Overall,

(15) $$\begin{array}{ccc} \frac{\partial E{\left(\Phi \right)}_{t}}{\partial h_{t}^{\left(1\right)}} & = & \left(h_{t}^{\left(1\right)}-h_{t}^{\left(L\right)}\right)-\left(h_{t}^{\left(1\right)}-h_{t}^{\left(L\right)}\right)D^{\left(L\right)}W^{\left(L-1\right)}D^{\left(L-1\right)}W^{\left(L-2\right)}\times \ldots \times D^{\left(2\right)}W^{\left(1\right)}. \end{array}$$

As a result, a defect $h_{t,d}^{\left(1\right)}$ is updated via Equation (12) with several iterations required to repair it.

## 4. Experiment 1: Feature Extraction

The purpose of this experiment is to verify the ability of the DSAE to extract the time-series of latent features. Driving behavior data were obtained from multiple sensors with an actual vehicle. In this experiment, the DSAE extracts the time-series of latent features from different datasets by preparing 12 datasets, D1 to D12, that include part or all of the measured sensor time-series data. PCA was also used as a comparative method. In the experiment, we evaluated the reconstruction error and the extracted latent features by using the two models. Additional details of the experiments are provided in the following subsections.

### 4.1. Experimental Conditions

We asked a participant to drive the vehicle through two types of courses shown in Figure 1. Pedestrians, other moving vehicles, and parked vehicles, were found along the courses. The vehicle traveled each course five times to obtain the driving behavior data. The data of circuits 1–5 corresponded to the first course, and data of circuits 6–10 corresponded to the second course, as shown in Figure 1. In total, we measured 12,958 frames of driving behavior data with a frame rate of 10 fps. Each frame of data included nine kinds of sensor information, which are listed in Table 1.

We considered the physical meaning of these nine kinds of sensor information. For the discussion in this paper, we divided the sensor information into three categories for reference. The sensor information measured for the accelerator-opening rate, brake master-cylinder pressure, and longitudinal acceleration were considered to be related to the acceleration of the vehicle; the sensor information obtained for the speed of the wheels, and the meter readings of the velocity and engine speed were considered to relate to the velocity of the vehicle; the steering angle can represent the change in the driving direction; and the longitudinal acceleration and yaw rate contained information about the acceleration of the vehicle and the change in the driving direction. Therefore, we assumed that the nine kinds of sensors information were generated from the latent features of the driving behavior, which were related to the velocity of the vehicle (*V*), the acceleration of vehicle (*A*), and the change in driving direction (*D*). Note that we did not demonstrably affirm the acceleration, velocity, and the change in the driving direction to be latent features of the driving behavior; instead, we assumed that the latent features were related to them.

Twelve datasets (D1 to D12) composed of different measured sensor information, as listed in Table 2, were prepared for the purpose of verifying and comparing the feature extraction performance. In Table 2, the included sensor information $I_{1}$ to $I_{9}$ are marked by “√” for each dataset; and “∘” shows the assumed latent features *V*, *A*, and *D* relating to each dataset. For example, dataset D1 included the accelerator opening rate and brake master-cylinder pressure, and was assumed to involve latent features in relation to *A*. D5 included the steering angle and speed of wheels that were assumed to involve latent features both in relation to *V* and *D*. Meanwhile, we assumed that D7 to D12 involved all the latent features in relation to *A*, *V*, and *D*.

We employed 12 PCAs and 12 DSAEs to extract the three-dimensional time-series of the latent features from D1 to D12 independently. The experimental results of [22] showed that, when using window size w=10, the extracted three-dimensional time-series of the latent features could represent a variety of driving behaviors and the transformations between driving behaviors by driving behavior visualization. Thus, for each PCA and DSAE, we set the time window size to w=10 (one second) for windowing processing. The PCAs extracted three-dimensional time-series of the latent features from the windowing data of each dataset, of which the structure is shown in the last column of Table 2. When DSAEs were used to extract three-dimensional data from each dataset, the dimensions of each layer were approximately half of the dimensions of the upper layer. The structure of the encoder of each DSAE is shown in the third column of Table 2. The set of parameters for the DSAEs is as follows: α=0.03, β=0.7, and ω=0.5, which were set based on the experience we gained during many previous experiments.

### 4.2. Evaluation of Model Training via Data Reconstruction

In our view, reconstructing driving behavior data from the extracted time-series of latent features is important for evaluating the feature extraction model, especially for the DSAE, which extracts the time-series of latent features by minimizing the reconstruction error with an encode-decode process. Meanwhile, data reconstruction is also important for repairing the defects in sensor time-series data because we can use it to compensate for the defects. In this regard, PCA is similar to an autoencoder in that it can be considered as an autoencoder model with a linear active function [30]. Therefore, PCA can reconstruct the input data by

(16) $$\begin{array}{c} h_{t}^{\left(L\right)}=W_{E}^{T}\left(W_{E}h_{t}^{\left(1\right)}\right), \end{array}$$

where $W_{E}\in R^{D_{H^{\left(1\right)}}\times D_{H^{\left(m\right)}}}$ is a matrix of eigenvectors. We reconstructed the windowing time-series data by decoding the time-series of latent features by using PCA and DSAE. The square error between the windowing time-series data and the reconstructed data can be used as a measure to evaluate the effectiveness of training. Considering the different dimensionalities of the data in D1 to D12, we used the average of the square error as an evaluation measure.

**Figure 4:**
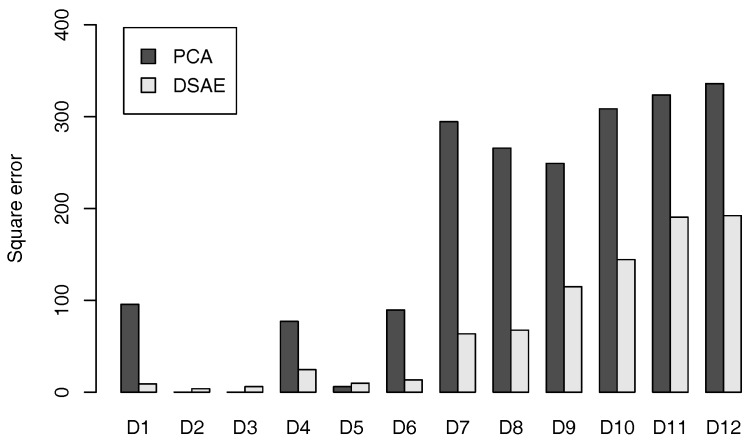
Averaged square error between windowing time-series data and reconstructed time-series data of datasets D1 to D12.

The averaged square error for D1 to D12 by using PCA and DSAE is shown in Figure 4. We can see that, when using the DSAEs, the square error between the windowing time-series data and the reconstructed time-series data was generally smaller than when using PCAs. Although the square errors of D2, D3, and D5 were smaller when using PCAs than when using DSAEs, these errors were insignificantly small regardless of whether PCAs or DSAEs were used. The above results showed that DSAE was more effective to reconstruct windowing time-series data from the time-series of latent features than PCA.

### 4.3. Evaluation of Latent Feature Extraction of Time-Series Using CCA

**Figure 5:**
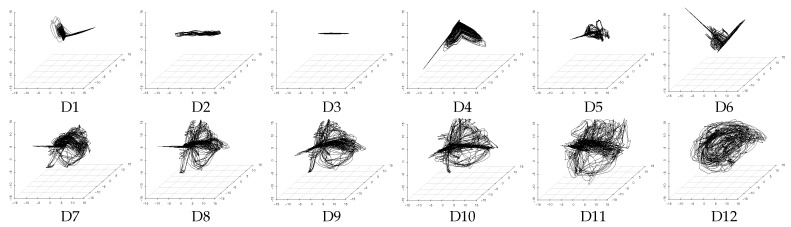
Three-dimensional time-series of latent features extracted from datasets D1 to D12 by PCA in the three-dimensional feature space.

**Figure 6:**
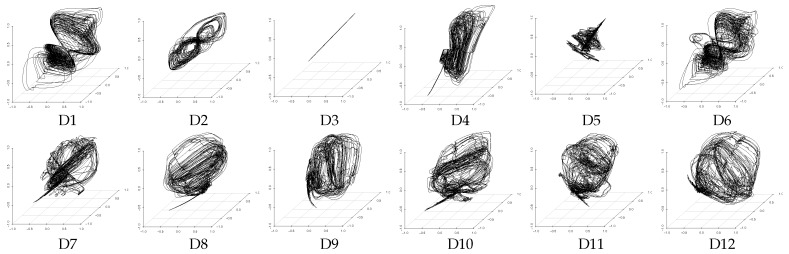
Three-dimensional time-series of latent features extracted from datasets D1 to D12 by DSAE in the three-dimensional feature space.

We consider that, if the method can obtain a similar extraction result from different datasets including the assumed three latent features of *V*, *A*, and *D*, then the extraction performance of this method is high. Here it is necessary to note that unsupervised learning methods, such as PCA and DSAE, extract time-series of latent features according to the distribution of the input data and generate the feature space automatically. This leads to a certain degree of freedom (the rotation and displacement of axes) between the feature spaces generated from the different datasets. For example, Figure 5 and Figure 6 show that the distributions of the extracted time-series of latent features from D7 to D12, which is expected to be similar, is rotated with the feature space generated by PCA and DSAE. Therefore, the standard correlation analysis could not be used to evaluate the experimental results directly because the time-series of latent features that were extracted from different datasets are not in the same space. Instead, we used canonical correlation analysis (CCA) [31] to analyze the correlation as a method to evaluate the similarity between the data from the two different spaces. CCA seeks a pair of linear transformations, one for each dataset, such that when the dataset is transformed, the corresponding coordinates are maximally correlated. The two optimized linear transformations reduce the influence of the degree of freedom. This process enables us to evaluate the correlation with a pair of extracted time-series of latent features. We use the canonical correlation coefficient as the evaluation measure to present the correlation. The range of the canonical correlation coefficient is [0,1]. As all the canonical correlation coefficients approach 1, the strength of the correlation between the two datasets increases. Likewise, if all the canonical correlation coefficients are close to 0, then the two datasets are uncorrelated. In both of these cases, both datasets are transformed using two optimized vectors.

We considered all pairs of datasets from D1 to D12, and used CCA to evaluate the correlation of features extracted from these pairs. The relationship between the time-series of latent features extracted from different datasets was evaluated by defining the distance of correlation (DOC) using the canonical correlation coefficients; in particular,

(17) $$\begin{array}{c} DOC=1-\frac{1}{D_{H}^{\left(m\right)}}\sum_{k=i}^{D_{H}^{\left(m\right)}}CCC_{k}, \end{array}$$

where $CCC_{k}$ is the *k*-th canonical correlation coefficient, and $D_{H}^{\left(m\right)}$ is the dimensionality of the extracted time-series of latent features; for this evaluation, $D_{H}^{\left(m\right)}=3$. If the time-series of latent features extracted from two datasets have a strong correlation, DOC is close to 0; conversely, DOC is close to 1.

**Figure 7:**
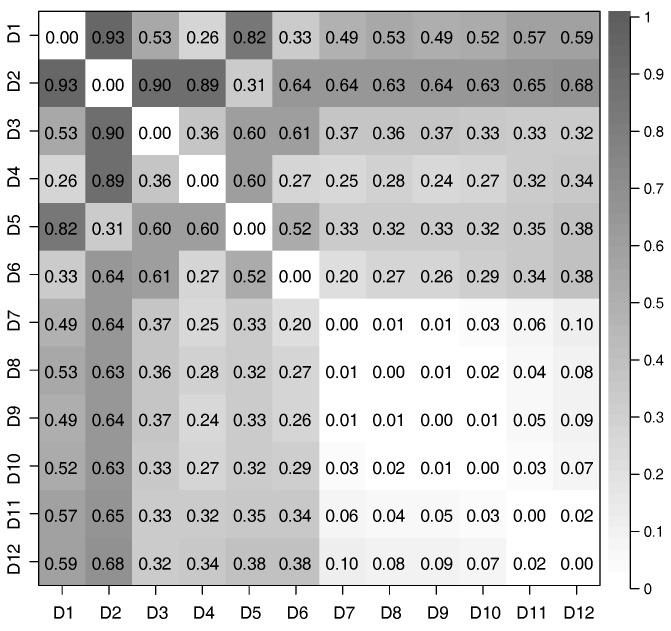
Distance of correlation between each pair of time-series of latent features extracted from the 12 datasets by using PCAs.

**Figure 8:**
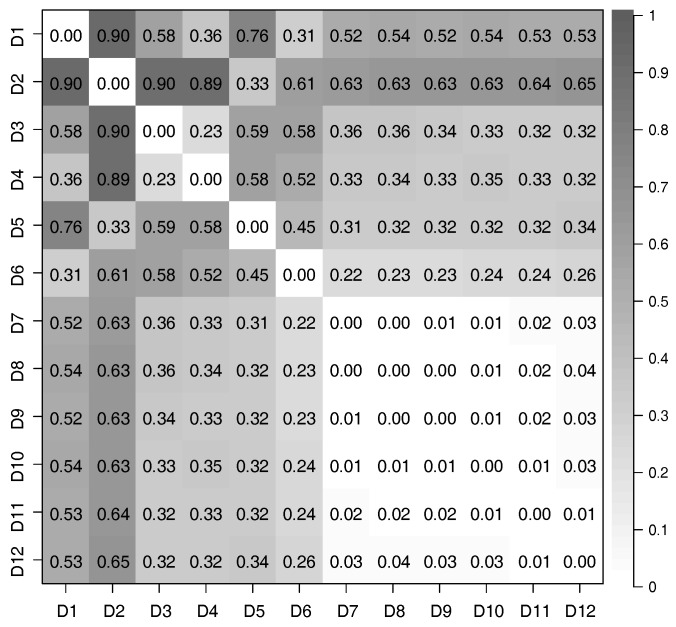
Distance of correlation between each pair of time-series of latent features extracted from the 12 datasets by using DSAEs.

**Figure 9:**
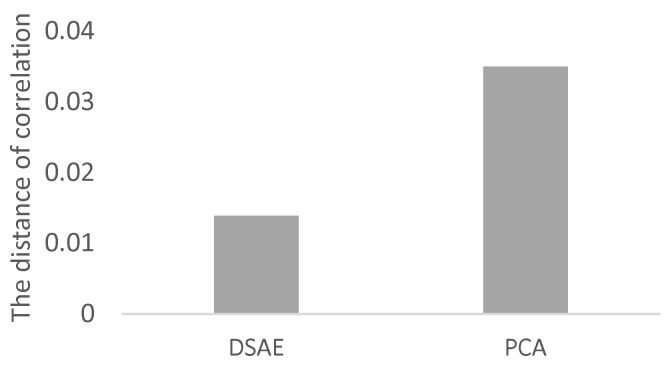
Averaged distance of correlation among pairs of time-series of latent features extracted from D7 to D12 by using PCAs and DSAEs.

We used Equation (17) to evaluate the correlation between each pair of time-series of latent features extracted from the 12 datasets we prepared. The distances of correlation among the time-series of latent features extracted from D1 to D12 by using the PCAs and DSAEs are shown in Figure 7 and Figure 8. In both figures, the distances of correlation among the extracted time-series of latent features from D1 to D6 were larger than 0.2. This result is in agreement with our assumptions, namely that D1 to D6 did not involve the same assumed latent features shown in Table 2. Meanwhile, we assumed D7 to D12 to involve the same latent features related to *V*, *A*, and *D*, which are also shown in Table 2. Therefore, we considered that the DOCs among the time-series of latent features extracted from D7 to D12 should be as small as possible. This assumption is verified by using the PCAs and DSAEs because the DOCs among the time-series of latent features extracted from D7 to D12 were less than 0.11. However, in Figure 7, the DOCs among the time-series of latent features extracted by using PCAs from D7 to D12 were larger than by using DSAEs. Particularly, when using PCA, the DOCs among D7-D11, D7-D12, and D9-D12 were obviously larger than when using DSAE. Finally, we measured the average value of DOCs among D7 to D12 by using both PCAs and DSAEs. The result is shown in Figure 9. The figure shows that, in contrast to PCA, the average DOC of DSAE was more than 2.5 times lower than that of PCA. In summary, we verified the ability of DSAE to extract the time-series of latent features from the driving behavior datasets with various degrees of redundancy, and especially that it outperformed PCA.

## 5. Experiment 2: Reducing the Negative Effect of Defects for Feature Extraction

The purpose of this experiment is to verify that our proposed method can reduce the negative effect of defects on the feature extraction of driving behavior. The sensor time-series data obtained when measuring actual driving behavior often includes defects. First, we manually created defects in different non-defective sensor time-series datasets. Second, we used a DSAE that was trained on non-defective sensor time-series data to repair the defects, and extracted the time-series of latent features from the repaired time-series data. Finally, we evaluated the ability to reduce the negative effect of defects with two criteria: the similarity between the repaired data and the non-defective data, and the similarity between latent features from the repaired data and the non-defective data.

### 5.1. Experimental Conditions

We assumed that each sensor-measured time-series dataset includes a periodical defect. Therefore, we synthetically introduced some defects into dataset D12 listed in Table 2, which included nine kinds of intact sensor time-series data. Based on D12, we prepared eight datasets, C1 to C8, which included defects in different sensor time-series data. However, we coordinated the time of the defects of longitudinal and lateral acceleration in C7, because the same acceleration sensor measured both of them. Details of those datasets are presented in Table 3. Non-defective sensor time-series data are shown as “√”, and sensor time-series data with defects are shown as “(√)”. Apparently, a defect persisting for a short period is easier to repair by linear interpolation because some of the driving behavior changes linearly in a short period, for example when the driver turns the steering left with uniform angular velocity. We consider our proposed method to be effective for defects that persist for long periods. Therefore, we created defects of 100 frames (10 s) every 300 frames (30 s) on D12. In this way, we introduced 4620 defective frames in the sensor time-series data.

We expected the different settings of the defect value to affect the model in different ways. For example, the defect values of −1 and 1 are the extremes for types of sensor time-series data of which the average is near 0 when the dataset is normalized to a range of [−1,1], such as in the case of the steering angle, longitudinal and lateral acceleration, and yaw rate. Meanwhile, there are types of sensor time-series data that include many values of −1, such as the accelerator opening rate and the brake master-cylinder pressure. For these cases, the defect value of −1 is less extreme than defect values of 1 and 0. Therefore, we set three types of defect values: 1, 0, −1.

### 5.2. Evaluation of Data Repair of Sensor Time-Series Data

This subsection describes our verification and comparison of the abilities of DSAE-FP and DSAE-BP to repair defects. We iteratively updated DSAE-FP 500 times to repair defects. Meanwhile, DSAE-BP was updated 2000 times with a learning rate of ψ=0.01 to repair the defects of each dataset in the series C1 to C8.

**Figure 10:**
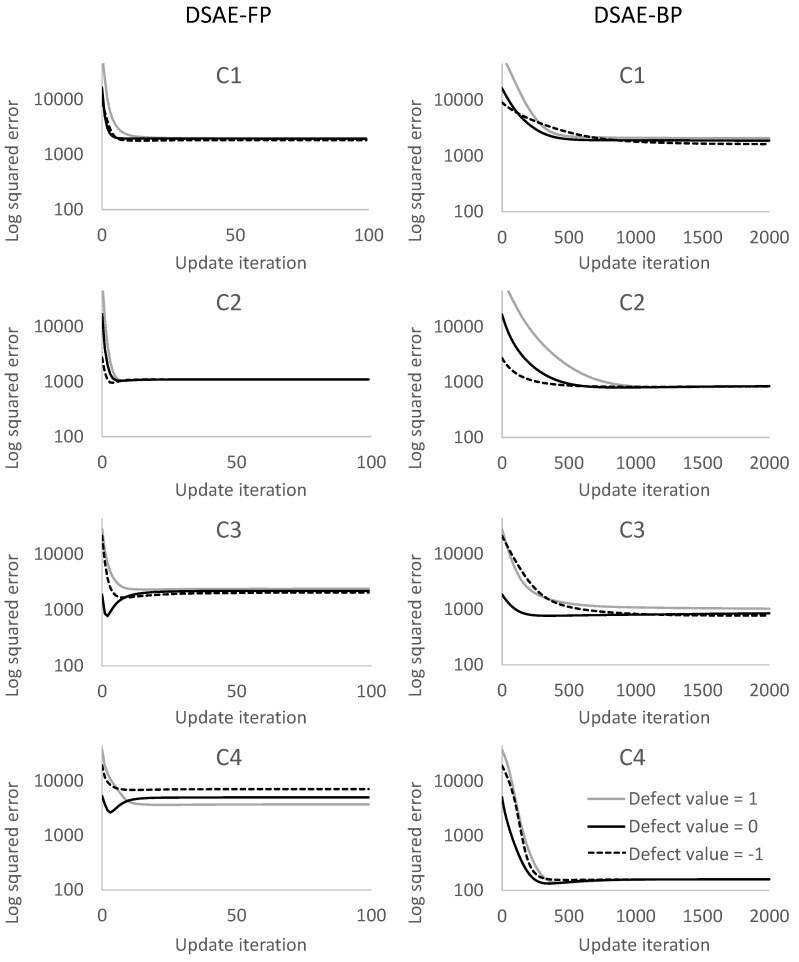
Square error between the repaired sensor time-series data and non-defective sensor time-series data for C1 to C4 at each update iteration. The plots in the columns on the left and right represent the results obtained by using DSAE-FP and DSAE-BP, respectively.

**Figure 11:**
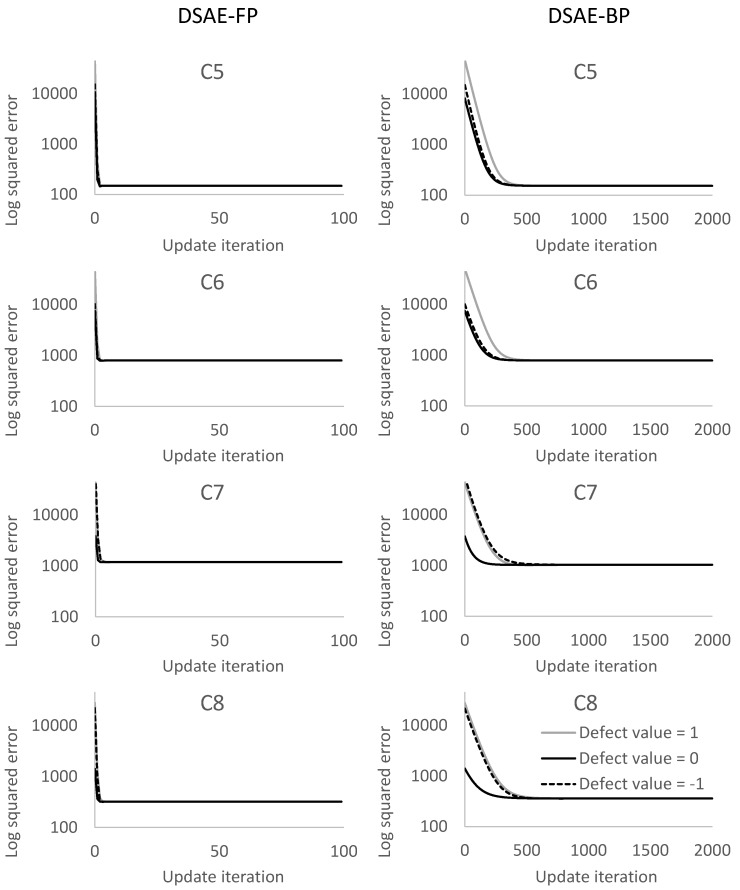
Square error between the repaired sensor time-series data and non-defective sensor time-series data for C5 to C8 at each update iteration. The plots in the columns on the left and right represent the results obtained by using DSAE-FP and DSAE-BP, respectively.

**Figure 12:**
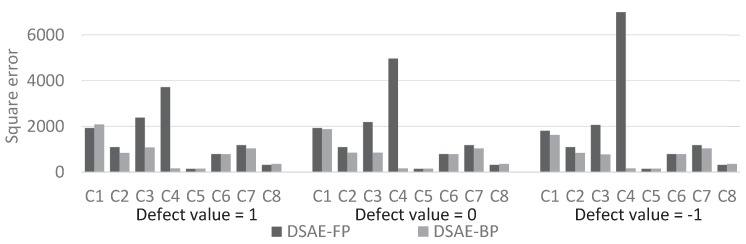
Convergence values of squared error with different initial value for each of the datasets when DSAE-FP and DSAE-BP were used.

**Figure 13:**
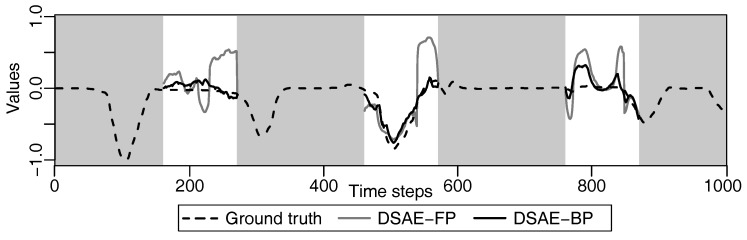
Example of defect repair of the steering angle by DSAE-FP and DSAE-BP for a part of C3 in the time-series, when the defect value was 1. A white background indicates the period of defects. The values in parentheses in the legend represent the defect values.

We calculated the square error between the updated defect value and the true value at each iterative cycle. Figure 10 and Figure 11 compare the results of using DSAE-FP and DSAE-BP. To display the convergence process of the square error more clearly, Figure 10 and Figure 11 only show the first 100 iterative cycles of the update for results of DSAE-FP. Note that the convergence speed is controlled by the learning rate when DSAE-BP is used, which means that a comparison of the convergence speed between DSAE-FP and DSAE-BP would have no significance. Therefore, we focused on the converged value of the square error. Figure 10 and Figure 11 show that the square error of each dataset was reduced and converged by using DSAE-FP and DSAE-BP, irrespective of whether the initial defect value was 1, 0, or −1. Except for C4, for the other results obtained with DSAE-FP the square error of the initial defect value of 1, 0, and −1 converged to similar values. This indicated that DSAE-FP repaired the defect value to a convergence value, but DSAE-FP easily enabled the defect values to converge to different local converged values if the initial defect values were different, such as the result of C4. In addition, Figure 10 shows that the square errors of C3 and C4 decreased before increasing, and finally converged when the initial defect value was set to 0. This also showed that the converged value is not necessarily the minimum of the square error in those cases when using DSAE-FP. Meanwhile, when we used DSAE-BP, the different initial values of the defect value can also be reduced and converge to a similar value. For most of the results, the converged values of the square error were small when using DSAE-BP compared to when using DSAE-FP, especially for C4. This result is also shown in Figure 12, which shows the converged values of the squared error with a different initial value for each dataset when DSAE-FP and DSAE-BP were used. To visualize the data repaired by DSAE-FP and DSAE-BP, Figure 13 shows part of the repaired sensor time-series data of the steering angle in C3 and the ground truth thereof when the defect value was 1. It shows that the repaired sensor time-series data using DSAE-BP were closer to the ground truth than when using DSAE-FP. In summary, the above experimental results show that DSAE-BP is more effective than DSAE-FP for repairing defective sensor time-series data of driving behavior.

### 5.3. Evaluation of Feature Extraction with Defective Data

We focused on feature extraction from datasets that include defective sensor time-series data. Especially, we evaluated the similarity of the time-series of latent features extracted from a dataset including defective and non-defective sensor time-series data. In the first evaluation experiment, we used the feature extraction methods PCA and DSAE without data repairing to show the negative effects of defect data on them. Second, we used the typical defect repair methods, linear interpolation (LI) and median filter (MF) with a window size of 100, before applying PCA or DSAE as the baseline to reduce the negative effect of defects on feature extraction. Note that LI and MF do not depend on the defect value because they use the data before and after the interval of defects to repair defects alone. Third, we used our proposed method DSAE-BP to extract the latent features from defective and non-defective sensor time-series data. We also used PCA-FP, PCA-BP and DSAE-FP as comparative methods. When we used PCA-FP, the defective sensor information was updated by its reconstructed data via PCA by Equation (16). Meanwhile, PCA-BP used the same defect-repairing algorithm as DSAE-BP, to update the defective element $h_{t^{\left(1\right)},d}$ in the sensor time-series data via Equation (12), but $\frac{\partial E{\left(\Phi \right)}_{t}}{\partial h_{t}^{\left(1\right)}}=\left(h_{t}^{\left(1\right)}-h_{t}^{\left(L\right)}\right)-\left(h_{t}^{\left(1\right)}-h_{t}^{\left(L\right)}\right)\left(W_{E}W_{E}^{T}\right)$.

We consider C1 to C8 to be the defective datasets based on D12. Meanwhile, the time-series of latent features of C1 to C8 were extracted through a trained model by using D12 to ensure that the feature spaces of C1 to C8 and D12 were the same. This enabled us to directly calculate the square error between the time-series of latent features extracted from defective and non-defective sensor time-series data to evaluate the negative effect on the extracted time-series of latent features. However, the feature spaces generated by PCA and DSAE were different, particularly their scales. Thus, the use of the square error to directly evaluate PCA and DSAE would not be sensible. Therefore, we used the coefficient of determination ($R^{2}$) [32] to evaluate the latent feature extraction of time-series by PCA and DSAE with and without the defects. This is because we return this evaluation as a regression problem, which is the time-series of latent features of D12 as the response variable, C1 to C8 as the explanatory variable, and the time-series of latent features extracted from C1 to C8 as the predicted values. We also considered the periods without defects in defective sensor time-series data to be the same as the corresponding periods of data in non-defective time-series data. Therefore, the time-series of latent features extracted from those periods were also the same, because the transformations of latent feature extraction are the same. To evaluate the negative effects caused by the defects only, we defined a sub-dataset $S\subset H^{\left(m\right)}$, which included the extracted time-series of latent features in the periods of defects, where $S\in R^{D_{H^{\left(m\right)}}\times N_{S}}$ and $N_{S}$ is the number of frames of defects, i.e., 4620. We defined $R^{2}$ (see Appendix A), which can be regarded as an opposite manifestation of the square error normalized by the variance of $H^{\left(m\right)}$ of D12. When the value of $R^{2}$ closely approximates 1, the time-series of latent features extracted from defective and non-defective sensor time-series data are similar. Conversely, the similarity is poor when $R^{2}$ is reduced. Especially when the time-series of latent features extracted from defective and non-defective sensor time-series data are very different, the numerator of the second term will be greater than the denominator in Equation (A1), thus $R^{2}$ will become a negative number.

The evaluation results of datasets C1 to C8 are presented in Table 4. The highest value of $R^{2}$ is presented in bold font and the second highest value is underlined for each defect value. First, to show the negative effects of defects on feature extraction, we used PCA and DSAE without defect repair to extract the latent features from defective and non-defective sensor time-series data. Table 4 shows that many of the values of $R^{2}$ were negative when the PCA and DSAE were used with defect values 1 and −1. When the defect value was 0, the values of $R^{2}$ were below 0.75 when PCA was used for C2 and DSAE was used for C1 and C2. This shows that the negative effect of defects on feature extraction was considerable. Second, we additionally used LI and MF before PCA and DSAE as the baseline to reduce the negative effect of defects on the feature extraction. Table 4 shows that all the values of $R^{2}$ were higher than the direct use of feature extraction when the LI and MF were used before the PCA and DSAE, regardless as to whether the defect value was 1, 0 or −1. Even though LI and MF could be used for this task, the values of $R^{2}$ for some of the datasets were below 0.9, e.g., C7 by LI and MF with PCA; C1 and C7 by LI with DSAE; and C1 and C4 by MF with DSAE. Third, we used our proposed method DSAE-BP and the comparative methods PCA-FP, PCA-BP, and DSAE-FP to extract the latent features from defective and non-defective sensor time-series data. Table 4 also shows that both the DSAE-FP and the DSAE-BP could update the defective sensor information to enable it to converge to a similar value, regardless of whether the defect value was selected as 1, 0, or −1. Meanwhile, most of the $R^{2}$ values of PCA-FP were negative. It is noteworthy that, when we evaluated $R^{2}$ of C7 and C8 with the defect values 1, 0, and −1 by using PCA-FP, the $R^{2}$ values became “–" that means the defective sensor information was diverged via update. That is, even though the FP method was used before the PCA, the effect of defects on the extracted latent features by PCA-FP was huge. DSAE-FP also used the FP method but its $R^{2}$ values were much higher than PCA-FP. Almost all of the $R^{2}$ values of DSAE-FP were higher than 0.9. However, for C4, the $R^{2}$ values of DSAE-FP were lower than 0.7 when the defect values were 1, 0, and −1. Table 4 shows that when we used PCA-BP and DSAE-BP, the $R^{2}$ values were higher than 0.95 for all the datasets and all the defect values. This shows that the use of the BP method to repair defective data was effective. In addition, the values presented in bold font in Table 4 shows that the highest value of $R^{2}$ was obtained when using DSAE-BP for most cases in this experiment, irrespective of whether the defect value was 1, 0 or −1.

Even if DSAE-BP did not achieve the highest value, its performance was the second best, as is evident from the underlined values in Table 4. In summary, the experimental results showed that the DSAE-BP can extract time-series of latent features that are similar to the real values when the sensor time-series data are defective. In other words, DSAE-BP can reduce the negative effect of defects on extracted time-series of latent features and it is superior to other comparative methods.

## 6. Application: Driving Behavior Segmentation with Defects

After verifying that DSAE-BP can reduce the negative effect on feature extraction, we show an example of its application to capture transitions in patterns of driving behavior and segment time-series data into segments. We either considered the defects in the time-series data block or altered the transitions in the patterns of driving behavior. Therefore, defects adversely affect the driving behavior segmentation to a large extent. We propose to use DSAE-BP, which is expected to ensure that the segmentation results of defective and non-defective sensor time-series data are similar.

In this experiment, we paired the time-series of latent features extracted from each dataset of C1 to C8 (including defects) with dataset D12 (that does not include defects). To show the effectiveness of the feature extraction method with the different defect values, 1, 0, −1, we also used the nine-dimensional normalized raw data (RAW) of C1 to C8 and D12 without the windowing proses to perform the segmentation task as a baseline. Meanwhile, we also used PCA, DSAE, PCA-BP, and DSASE-FP as comparative methods. Note that, we excluded PCA-FP as a comparison method because of its poor ability to extract latent features with defects in previous experiments. To segment the time-series of driving behaviors, we employed a sticky hierarchical Dirichlet process hidden Markov model (sticky HDP-HMM) [33], which was used for segmenting driving behavior data in previous studies [8,21,34,35]. The RAW data and time-series of latent features extracted from D12 were used to train the sticky HDP-HMM. Then the trained sticky HDP-HMMs were used to estimate the change points of segments from C1 to C8. Finally, we defined a segmentation distance (see Appendix B) to evaluate the similarity of segmentation between defective and non-defective sensor time-series data. If the segmentation distance is short, it means two segmentation results are similar.

In this experiment, we examined the segmentation distance between D12 and C1 to C8. We set the parameter *w* of the time window to four, which means the range of the time window included nine frames. We evaluated the segmentation distance for each dataset via each method 10 times because sticky HDP-HMM is a probability model that samples the boundaries of segments via a random process. The results of the average segmentation distance are presented in Table 5. The shortest average segmentation distance (best performance) is shown in bold font; the second shortest average segmentation distance is shown in underlined.

First, we focused on the performance of FP and BP with PCA and DSAE on different defect values. Table 5 shows that regardless of whether the defect value was set to 1, 0, or −1, the average segmentation distances of each dataset processed by using PCA-BP, DSAE-FP and DSAE-BP are more similar to each other than by using other comparative methods. Here, we take C1 as an example. The average segmentation distances of C1 by PCA-BP (153, 155, 152), DSAE-FP (132, 132, 131) and DSAE-BP (126, 123, 123) were more similar to each other than those by RAW (381, 325, 158), PCA (306, 252, 116), and DSAE (351, 310, 137) when the defect values were 1, 0, and −1. The reason is that the square error of each dataset with defect values of 1, 0, and −1 converged to a similar value when FP and BP were used with PCA and DSAE, which can be seen in Figure 10 and Figure 11 for DSAE-FP and DSAE-BP. However, this conclusion does not apply to C4 by using DSAE-FP (235, 328, 305) because the converged values of the square error were not similar by using DSAE-FP for C4 with the initial defect values of 1, 0, and −1 in the Section 5.2.

Next, we compared the performance of DSAE-BP with other comparable methods on different defect values, respectively. The results in Table 5 show that, when the defect value was set to 1, the average segmentation distance between the defective and non-defective sensor time-series data were shortest by using DSAE-BP with DSAE-FP in the second place, except C3 and C4. PCA-BP obtained the best performance on C3 and C4 and DSAE-BP got the second place. Meanwhile, when the defect value was set to 0, DSAE-BP was most effective in terms of achieving the shortest average segmentation distances between D12 and most of datasets C1 to C8. However, for C3, the result was poor in comparison. This reason is that the sensor time-series data of the steering angle in dataset C3 are defective. The measured sensor time-series data of steering angle included many values of 0, especially in the case of straight-line driving. Therefore, if the defect value of the steering angle was set to 0, then this defect value has a high probability to be equal to the true value. Moreover, when the defect value was set to −1, DSAE-BP achieved the shortest average segmentation distances for C2, C5, C6, C7, and C8, and it also achieved the second shortest average segmentation distances for C1, C3, and C4.

The above results showed that PCA-BP, DSAE-FP, and DSAE-BP have the ability to reduce the negative effect for driving behavior segmentation. Note that, DSAE-BP still outperformed PCA-BP because DSAE-BP could extract the latent features of time-series from defective that were more closer to the latent features extracted from non-defective sensor time-series data than when using PCA-BP. Meanwhile, DSAE-BP outperformed DSAE-FP because DSAE-FP easily enables the defect values to converge to fixed points but it does not guarantee that the fixed points are local optima.

## 7. Discussion for Advantages and Limitations of Proposed Method

In this study, we propose using DSAE-BP, that based on a trained DSAE, to repair the defective driving behavior data. The advantage of the proposed approach is that DSAE model is trained only one time and two tasks can be completed – latent feature extraction and defective data repairing. The representation of latent features is critical to both tasks. If the latent features of DSAE are difficult to represent one of the driving behaviors, then DSAE-BP will have trouble repairing the defective data when such driving behavior occurs. It is an important topic that is how to design a DSAE that can extract the latent features well. For now, optimizing the structure of the neural network remains an open question. In this study, we still use our experience from our previous study to design the structure of DSAE. In our previous study, we verified the usability of the designed DSAE model by a visualization method [22]. Although it is not sufficient to verify this structure is the best, the experimental results show that it is effective.

In addition, DSAE can be used for a variety of tasks as the pre-training such as driving behavior classification. We repaired the defective data by minimizing the square error in the experiment. However, in the view point of driving behavior classification, square error does not guarantee that the repaired data will not vibrate. For example, the right side of the period of defects in Figure 13 shows that the repaired sensor information of steering angle by DSAE-BP vibrated up and down on the ground truth. If this kind of repaired sensor information will use to classify driving behaviors, then it would lead to misclassification. For this problem, we suggest that DSAE can be fine-tuned for different tasks.

## 8. Conclusions

We propose a method for extracting low-dimensional time-series of latent features from multi-dimensional driving behavior data using a DSAE. In addition, the DSAE can also repair the defects in sensor time-series data in combination with back propagation (DSAE-BP). In the first experiment, we show that DSAE could extract highly correlated low-dimensional time-series of latent features by reducing the redundancy to various degrees in different multi-dimensional time-series data of driving behavior. Furthermore, we verify that DSAE-BP could repair the defective sensor time-series data and reduce the negative effect of defects on the extracted time-series of latent features in second experiment. We also show in the third experiment that, based on the latent features extracted by DSAE-BP, the negative effect of defects on the driving behavior segmentation task could be reduced.

The proposed defect-repairable latent feature extraction method has a wide range of applications, e.g., the prediction, visualization, segmentation, and estimation of the latent structure of driving behaviors. Many types of data-driven driving behavior analysis methods have been proposed. Most of them benefited from low-dimensional and informative feature vectors. First, by reducing the dimension of feature vectors by retaining the information contained within the time-series data, we can reduce the computational cost of the post-process, e.g., prediction or segmentation. Second, appropriate feature vectors also increase the generalization performance of the post-process. We show that our proposed method can reduce the dimensionality by retaining the contained information and reducing the redundancy (see Experiment 1). Practically, feature extraction needs to be robust to defects and outliers for its application. Our method can reduce the adverse effect of defects significantly (see Experiment 2). These favorable functions will increase the performance of various applications including driving behavior segmentation (see Experiment 3). We believe that the method has an impact on data-driven driving behavior analyses and contributes to the safety of driving.

In this study, DSAE completed two tasks when only trained once: (1) extracting time-series of latent features; and (2) repairing defects in sensor time-series data by using a back-propagation method. The gradient information for DSAE-BP may also be used to detect the defects of driving behaviors. Based on this idea, it may be possible to detect the occurrence area of defects. However, it is difficult to accurately determine which of the measured sensor information is defective. The reason for this problem is that a variety of sensor information has been fused in the middle layer of the DSAE. How to solve this problem is still an open question. In addition, other unsupervised feature extraction methods would be compared with DSAE such as variational autoencoder [36] and long short term memory [37] for extracting latent features of driving behaviors in the future.

## Figures and Tables

**Table 1:** Measured sensor information and the assumed latent features of driving behavior.

**Measured Sensor Information**		
$I_{1}$: Accelerator opening rate	$I_{2}$: Brake master-cylinder pressure	$I_{3}$: Steering angle
$I_{4}$: Speed of wheels	$I_{5}$: Meter readings of velocity	$I_{6}$: Engine speed
$I_{7}$: Longitudinal acceleration	$I_{8}$: Lateral acceleration	$I_{9}$: Yaw rate
**Assumed latent features**		
*V*: The feature is related to the velocity		
*A*: The feature is related to the acceleration		
*D*: The feature is related to a change in the driving direction		

**Table 2:** Sensor information included in the 12 datasets we prepared and the PCA and DSAE designs for each of them.

Data Sets	Included Sensor Information									Assumed Latent Features			Encoder Structure of DSAE (with Window Size: 10)	The Structure of PCA (with Window Size: 10)
	$I_{1}$	$I_{2}$	$I_{3}$	$I_{4}$	$I_{5}$	$I_{6}$	$I_{7}$	$I_{8}$	$I_{9}$	V	A	D		
D1	√	√									∘		2D×10=20D→10D→5D→3D	2D×10=20D→3D
D2			√									∘	1D×10=10D→5D→3D	1D×10=10D→3D
D3				√						∘			1D×10=10D→5D→3D	1D×10=10D→3D
D4	√	√		√						∘	∘		3D×10=30D→15D→7D→3D	3D×10=30D→3D
D5			√	√						∘		∘	2D×10=20D→10D→5D→3D	2D×10=20D→3D
D6	√	√	√								∘	∘	3D×10=30D→15D→7D→3D	3D×10=30D→3D
D7	√	√	√	√						∘	∘	∘	4D×10=40D→20D→10D→5D→3D	4D×10=40D→3D
D8	√	√	√	√	√					∘	∘	∘	5D×10=50D→25D→12D→6D→3D	5D×10=50D→3D
D9	√	√	√	√	√	√				∘	∘	∘	6D×10=60D→30D→15D→7D→3D	6D×10=60D→3D
D10	√	√	√	√	√	√	√			∘	∘	∘	7D×10=70D→35D→17D→8D→3D	7D×10=70D→3D
D11	√	√	√	√	√	√	√	√		∘	∘	∘	8D×10=80D→40D→20D→10D→3D	8D×10=80D→3D
D12	√	√	√	√	√	√	√	√	√	∘	∘	∘	9D×10=90D→45D→22D→11D→3D	9D×10=90D→3D

**Table 3:** Sensor time-series data included in eight prepared datasets.

Data Sets	Included Sensor Time-Series Data								
	$I_{1}$	$I_{2}$	$I_{3}$	$I_{4}$	$I_{5}$	$I_{6}$	$I_{7}$	$I_{8}$	$I_{9}$
C1	(√)	√	√	√	√	√	√	√	√
C2	√	(√)	√	√	√	√	√	√	√
C3	√	√	(√)	√	√	√	√	√	√
C4	√	√	√	(√)	√	√	√	√	√
C5	√	√	√	√	(√)	√	√	√	√
C6	√	√	√	√	√	(√)	√	√	√
C7	√	√	√	√	√	√	(√)	(√)	√
C8	√	√	√	√	√	√	√	√	(√)

**Table 4:** $R^{2}$s between the extracted time-series of latent features of D12 and C1 to C8. The highest value of $R^{2}$ is presented in bold font; the second highest value is underlined for each defect value.

Defect Values	Mehods	C1	C2	C3	C4	C5	C6	C7	C8
1	LI+PCA	0.958	0.970	0.962	0.990	0.989	0.976	0.825	0.948
1	LI+DSAE	0.864	0.945	0.938	0.944	0.997	0.988	0.890	0.992
1	MF+PCA	0.950	0.952	0.957	0.951	0.945	0.976	0.893	0.960
1	MF+DSAE	0.855	0.916	0.933	0.725	0.989	0.990	0.924	0.995
1	PCA	0.581	−0.173	0.520	0.637	0.595	0.481	−0.269	0.174
1	DSAE	−0.233	−0.671	0.213	−0.791	0.916	0.729	0.579	0.874
1	PCA-FP	0.832	−1.01	−177	−0.858	−0.055	0.337	–	–
1	DSAE-FP	0.968	0.975	0.906	0.750	**1.00**	**0.997**	0.987	**0.999**
1	PCA-BP	**0.985**	0.975	**0.987**	**0.998**	0.996	0.989	0.953	0.986
1	DSAE-BP	0.964	**0.983**	0.974	0.992	**1.00**	**0.997**	**0.990**	**0.999**
0	LI+PCA	0.958	0.970	0.962	0.990	0.989	0.976	0.825	0.948
0	LI+DSAE	0.864	0.945	0.938	0.944	0.997	0.988	0.890	0.992
0	MF+PCA	0.950	0.952	0.957	0.951	0.945	0.976	0.893	0.960
0	MF+DSAE	0.855	0.916	0.933	0.725	0.989	0.990	0.924	0.995
0	PCA	0.904	0.745	0.970	0.949	0.930	0.925	0.912	0.961
0	DSAE	0.641	0.553	0.955	0.692	0.986	0.960	0.947	0.995
0	PCA-FP	0.832	−1.01	−177	−0.858	−0.055	0.337	–	–
0	DSAE-FP	0.968	0.975	0.916	0.653	**1.00**	**0.997**	0.987	**0.999**
0	PCA-BP	**0.985**	0.975	**0.987**	**0.998**	0.996	0.989	0.953	0.986
0	DSAE-BP	0.969	**0.983**	0.981	0.992	**1.00**	**0.997**	**0.990**	**0.999**
−1	LI+PCA	0.958	0.970	0.962	0.990	0.989	0.976	0.825	0.948
−1	LI+DSAE	0.864	0.945	0.938	0.944	0.997	0.988	0.890	0.992
−1	MF+PCA	0.950	0.952	0.957	0.951	0.945	0.976	0.893	0.960
−1	MF+DSAE	0.855	0.916	0.933	0.725	0.989	0.990	0.924	0.995
−1	PCA	0.949	0.961	0.642	0.814	0.870	0.901	−0.581	0.351
−1	DSAE	0.862	0.930	0.385	−0.080	0.976	0.954	0.430	0.904
−1	PCA-FP	0.832	−1.01	−177	−0.858	−0.055	0.337	–	–
−1	DSAE-FP	0.970	0.975	0.921	0.489	**1.00**	**0.997**	0.987	**0.999**
−1	PCA-BP	**0.985**	0.975	**0.987**	**0.998**	0.996	0.989	0.953	0.986
−1	DSAE-BP	0.975	**0.983**	0.982	0.992	**1.00**	**0.997**	**0.990**	**0.999**

**Table 5:** Average segmentation distances between D12 and C1 to C8 with defect values of 1, 0, and −1. The shortest average segmentation distance (best performance) is shown in bold font; the second shortest average segmentation distance is shown in underlined.

Defect Value	Methods	C1	C2	C3	C4	C5	C6	C7	C8
1	RAW	381	524	578	402	358	321	312	338
1	PCA	306	408	295	300	304	322	342	326
1	DSAE	351	424	332	344	226	288	325	277
1	PCA-BP	153	167	**98.4**	**87.9**	98.4	151	187	94.3
1	DSAE-FP	132	149	196	235	50.4	89.5	143	59.1
1	DSAE-BP	**126**	**125**	133	142	**46.3**	**81.8**	**132**	**56.5**
0	RAW	325	437	166	321	224	210	108	117
0	PCA	252	329	93.1	199	207	237	231	113
0	DSAE	310	352	126	281	137	213	196	72.6
0	PCA-BP	155	171	**92.3**	**90.0**	94.5	145	194	91.7
0	DSAE-FP	132	155	233	328	46.3	89.1	146	**56.4**
0	DSAE-BP	**123**	**123**	141	142	**43.6**	**84.2**	**132**	58.8
−1	RAW	158	177	352	347	260	203	344	317
−1	PCA	**116**	174	288	263	218	249	342	316
−1	DSAE	137	181	322	377	118	213	332	275
−1	PCA-BP	152	170	**93.5**	**88.3**	95.3	147	189	91.2
−1	DSAE-FP	131	154	228	305	44.1	88.1	144	57.0
−1	DSAE-BP	123	**125**	144	142	**43.8**	**82.9**	**129**	**55.7**

## Appendix A. Similarity between Two Extracted Time-Series of Latent Features via the Same Trained Model

To evaluate the similarity of time-series of latent features extracted from defective and non-defective sensor time-series data, we defined $R^{2}$, which can be regarded as an opposite manifestation of the square error normalized by the variance of time-series of latent features $H^{\left(m\right)}$ of D12 ( not including defects):

(A1) $$\begin{array}{c} R^{2}=1-\frac{\frac{1}{N_{S}}\sum_{i=1}^{N_{S}}{\left(s_{i}-{\tilde{s}}_{i}\right)}^{T}\left(s_{i}-{\tilde{s}}_{i}\right)}{\sigma^{2}}, \end{array}$$

where $s_{i}$ is the *i*-th vector of the sub-dataset *S* extracted from the sub-dataset of D12, and ${\tilde{s}}_{i}$ is the *i*-th vector of the sub-dataset *S* extracted from C1 to C8 (the sub-dataset $S\subset H^{\left(m\right)}$, which included the extracted time-series of latent features in the periods of defects). $\sigma^{2}\in R$ is the variance of $H^{\left(m\right)}$ of D12:

(A2) $$\begin{array}{c} \sigma^{2}=\frac{1}{N_{V}}\sum_{j=1}^{N_{V}}{\left(h_{j}^{\left(m\right)}-{\bar{h}}^{\left(m\right)}\right)}^{T}\left(h_{j}^{\left(m\right)}-{\bar{h}}^{\left(m\right)}\right), \end{array}$$

where ${\bar{h}}^{\left(m\right)}$ is a mean vector of $H^{\left(m\right)}$. When the value of $R^{2}$ is close to 1, it means that the time-series of latent features extracted from defective and non-defective sensor time-series data are similar. Conversely, the similarity is poor when $R^{2}$ is reduced. Especially when the time-series of latent features are extracted from defective and non-defective sensor time-series the data are very different, and then the numerator of the second term will be greater than the denominator in Equation (A1), hence $R^{2}$ will become a negative number.

## Appendix B. Similarity between Two Segment Results

To evaluate the similarity of the results of segmenting RAW data or extracted time-series of latent features from defective and non-defective sensor time-series data, we introduce an evaluative method in this subsection.

For example, $H^{\left(m\right)}\in R^{D_{H^{\left(m\right)}}\times N_{V}}$ is a matrix of the time-series of latent features extracted from non-defective sensor time-series data, and ${\tilde{H}}^{\left(m\right)}\in R^{D_{H^{\left(m\right)}}\times N_{V}}$ is a matrix of the time-series of latent features extracted from sensor time-series data with defects. We used a sticky HDP-HMM trained by using $H^{\left(m\right)}$ (non-defective) to infer their respective hidden states and sample the boundaries of segments many times. $u^{\left(l\right)}=\left(u_{1}^{\left(l\right)},u_{2}^{\left(l\right)},\ldots ,u_{k}^{\left(l\right)}\right)\in R^{k}$ is a binary vector that shows the boundaries of segments sampled from $H^{\left(m\right)}$ in *l*-time of sampling, where $k=N_{V}-1$ is the number of intervals. If the hidden state of a sticky HDP-HMM changes at the *i*-th interval, then $u_{i}^{\left(l\right)}=1$; else, $u_{i}^{\left(l\right)}=0$. The vector of the change point of ${\tilde{H}}^{\left(m\right)}$ in *l*-time of sampling is shown as $v^{\left(l\right)}$, which is calculated as well as $u^{\left(l\right)}$.

Next, we calculate the segment probability by counting the boundaries of a segment at each interval by sampling many times. A vector of the segment probability can be shown by $p=\left(p_{1},\ldots ,p_{m}\right)\in R^{m}$ for $H^{\left(m\right)}$, which is calculated by

(A3) $$\begin{array}{c} p=\frac{1}{K}\sum_{j=1}^{K}u^{\left(j\right)}, \end{array}$$

where *K* is the number of samples. Similarly, the vector of the segment probability for ${\tilde{H}}^{\left(m\right)}$ is represented by q, which is calculated by

(A4) $$\begin{array}{c} q=\frac{1}{K}\sum_{j=1}^{K}v^{\left(j\right)}. \end{array}$$

Finally, we evaluate the similarity of the segmentation for $H^{\left(m\right)}$ and ${\tilde{H}}^{\left(m\right)}$ by comparing p and q. We consider each sampling time to be a random process. Even if the real probability of the segment at the *i*-th interval is very high, a boundary of segment $u_{i}=1$ was not necessarily sampled at this interval. Therefore, we evaluate the similarity of $H^{\left(m\right)}$ and ${\tilde{H}}^{\left(m\right)}$ by using the range of the segment probability in [i−w,i+w]. In particular, we calculate the average of the segment probability ${\bar{p}}_{i}$ and ${\bar{q}}_{i}$ in the range by

(A5) $$\begin{array}{c} {\bar{p}}_{i}=\frac{1}{2w+1}\sum_{a=i-w}^{i+w}p_{a}, \end{array}$$

(A6) $$\begin{array}{c} {\bar{q}}_{i}=\frac{1}{2w+1}\sum_{a=i-w}^{i+w}q_{a}, \end{array}$$

where *w* is a time window and w<i<n−w. Finally, we define the segmentation distance between $H^{\left(m\right)}$ and ${\tilde{H}}^{\left(m\right)}$ based on the segment probability:

(A7) $$\begin{array}{ccc} D(\bar{p},\bar{q}) & = & \frac{1}{\sqrt{2}}\sum_{i=w+1}^{k-w}\sqrt{{\left({\bar{p}}_{i}-{\bar{q}}_{i}\right)}^{2}+{\left(\left(1-{\bar{p}}_{i}\right)-\left(1-{\bar{q}}_{i}\right)\right)}^{2}}\\ & = & \frac{1}{\sqrt{2}}\sum_{i=w+1}^{k-w}\sqrt{{\left({\bar{p}}_{i}-{\bar{q}}_{i}\right)}^{2}+{\left({\bar{q}}_{i}-{\bar{p}}_{i}\right)}^{2}}\\ & = & \frac{1}{\sqrt{2}}\sum_{i=w+1}^{k-w}\sqrt{2}|{\bar{p}}_{i}-{\bar{q}}_{i}\\ & = & \sum_{i=w+1}^{k-w}\left|{\bar{p}}_{i}-{\bar{q}}_{i}\right| \end{array}$$

If the segmentation distance is short, it means two segmentation results are similar.
